# Nutraceutical Properties of Olive Oil Polyphenols. An Itinerary from Cultured Cells through Animal Models to Humans

**DOI:** 10.3390/ijms17060843

**Published:** 2016-05-31

**Authors:** Stefania Rigacci, Massimo Stefani

**Affiliations:** Department of Biomedical Experimental and Clinical Sciences, University of Florence, Viale Morgagni 50, 50134 Florence, Italy; stefania.rigacci@gmail.com

**Keywords:** Mediterranean diet, olive oil, polyphenols, oleuropein, hydroxytyrosol, neurodegeneration, cardiovascular disease, type 2 diabetes, obesity, cancer, metabolic syndrome, NASH

## Abstract

The increasing interest in the Mediterranean diet hinges on its healthy and anti-ageing properties. The composition of fatty acids, vitamins and polyphenols in olive oil, a key component of this diet, is considered a key feature of its healthy properties. Therefore, it is of significance that the Rod of Asclepius lying on a world map surrounded by olive tree branches has been chosen by the World Health Organization as a symbol of both peace and well-being. This review travels through most of the current and past research, recapitulating the biochemical and physiological correlations of the beneficial properties of olive tree (*Olea europaea*) polyphenols and their derivatives found in olive oil. The factors influencing the content and beneficial properties of olive oil polyphenols will also be taken into account together with their bioavailability. Finally, the data on the clinical and epidemiological relevance of olive oil and its polyphenols for longevity and against age- and lifestyle-associated pathologies such as cancer, cardiovascular, metabolic and neurodegenerative diseases are reviewed.

## 1. Introduction

Humanity living in developed countries is experiencing an increase in life expectancy; however, this positive outcome seems to be at the cost of a greater incidence of lifestyle- and age-associated diseases. These include cardiovascular diseases (CVDs), cancer and amyloid pathologies, both systemic (e.g., type 2 diabetes, T2DM) and neurodegenerative (e.g., Alzheimer’s (AD) and Parkinson (PD) diseases). Most of these pathologies are particularly hard to treat due to their slow progression, possibly spanning several decades, and the appearance of their clinical signs at mid- or old age, when cell loss is already conspicuous and irreversible. It is then evident that, in the absence of early reliable diagnostic tools and effective therapies, prevention is still the best strategy to combat these pathological conditions.

It is not surprising that the shift of researchers’ attention from “cure” to “prevention” has gradually led to an extension of the focus of their search, by adding “food”, and hence “diet”, to “drugs”. Several epidemiological and observational studies support the belief that traditional alimentary regimens such as the Mediterranean (MD) and Asian diets are associated with improved ageing and a reduced incidence of age-associated diseases, including CVDs, cancer and cognitive decline [1]. The new design of the MD pyramid, proposed by the Mediterranean Diet Foundation Expert Group [2], emphasizes the importance, in addition to caloric restriction (CR), of frugality, conviviality, physical activity and adequate rest; it also confirms the importance of the plant-based core (vegetables, fruits, legumes, grains, nuts and seeds) and, in particular, of extra virgin olive oil (EVOO) as the main lipid source. Moreover, a key feature of the MD is the high intake of phytonutrients (notably vitamins and natural phenols) that, by themselves, can induce multiple signalling pathways involved in protein homeostasis, DNA repair, metabolism regulation and antioxidant defences that recall a caloric restriction regime [3,4].

A key feature of natural phenolics is their remarkable antioxidant power. The latter has been associated with many beneficial properties of plant polyphenols via modulation of oxidative pathways [2], through direct action on enzymes, proteins, receptors and several types of signalling pathways [3,4] as well as by interfering with epigenetic modifications of chromatin [5]. In particular, the beneficial effects of olive oil and olive leaf extracts were already known in the ancient world, and scientifically investigated since the last couple of centuries, leading to a focus on their biological properties, including the antioxidant, antimicrobial, hypoglycemic, vasodilator and antihypertensive effects, whose clinical significance was first reported in 1950 [6]. Some of these properties have led to the inclusion in the European Pharmacopoeia (Ph. Eur.) of the 80% alcoholic extract of olive leaves [7], containing oleuropein (OLE), hydroxytyrosol (HT), caffeic acid, tyrosol, apigenin and verbascoside [8].

The increasing interest in natural polyphenols has produced a plethora of studies that have investigated their medical efficacy *in vitro*, in cell cultures, in model organisms and, to a lesser extent, in humans, together with the biochemical and biological modifications underlying their effects. Plant polyphenols, or their molecular scaffolds, can also be the starting point in developing new drugs especially designed to combat chronic inflammatory states, atherosclerosis and the risk of thrombosis related to CVDs [9], cancer [10], amyloid deposition associated with AD and T2DM, and age-associated neurodegeneration [1,11].

Here we review the results of the studies on the polyphenols found in the olive tree and in the EVOO and the most recent advances towards their possible clinical use, mainly concerning neurodegenerative diseases, atherosclerosis, cancer, T2DM and the metabolic syndrome.

## 2. Olive Tree Polyphenols

Natural phenolic substances are secondary plant metabolites, a major group of plant compounds (over 8000) chemically characterized by the presence of one or more aromatic rings with one or more hydroxyl substituents [1]. Plant polyphenols are elaborated as phytoalexins used to combat pests and bacterial infections. The olive tree (*Olea europaea*) produces its own battery of polyphenols that includes flavonols, lignans and glycosides. The latter belong to the class of iridoids, a type of monoterpenes composed of a cyclopentane ring fused to a six-atom oxygen heterocycle; the molecules containing a broken cyclopentane ring are known as secoiridoids.

Olive tree polyphenols are found in the lipid and water (as minute droplets), fractions of olive oil, and include the phenolic alcohols, HT (3,4-dihydroxyphenylethanol, 3,4-DHPEA) and tyrosol (*p*-hydroxyphenylethanol, *p*-HPEA) and their secoiridoid precursors. These include the HT ester of elenolic acid (known as oleuropein, OLE), the main responsible for the bitter taste of olive leaves and drupes; the dialdehydic derivative of decarboxymethyl elenolic acid bound to either HT (3,4-dihydroxyphenylethanol-elenolic acid dialdehyde, 3,4-DHPEA-EDA, also known as oleacein) or to tyrosol (*p*-hydroxyphenylethanol-elenolic acid dialdehyde, *p*-HPEA-EDA, also known as oleocanthal). The latter is the main responsible for the burning sensation that occurs in the back of the throat when consuming EVOO [12,13]. Olive tree polyphenols also include verbascoside, the caffeoylrhamnosylglucoside of HT, a phenolic acid derivative, the lignans 1-acetoxypinoresinol and pinoresinol, and other secoiridoids.

Olive tree polyphenols may be responsible for some of the properties of medical interest in this plant; these include anti-atherogenic, antihepatotoxic, hypoglycemic, anti-inflammatory, antitumor, antiviral and immunomodulator activities [14,15] that appear only in part related to the antioxidant power of these molecules. OLE, demethyloleuropein and ligstroside, together with their metabolic derivatives (elenolic acid, HT), are the most abundant phenolics in the EVOO [16].

**Figure 1:**
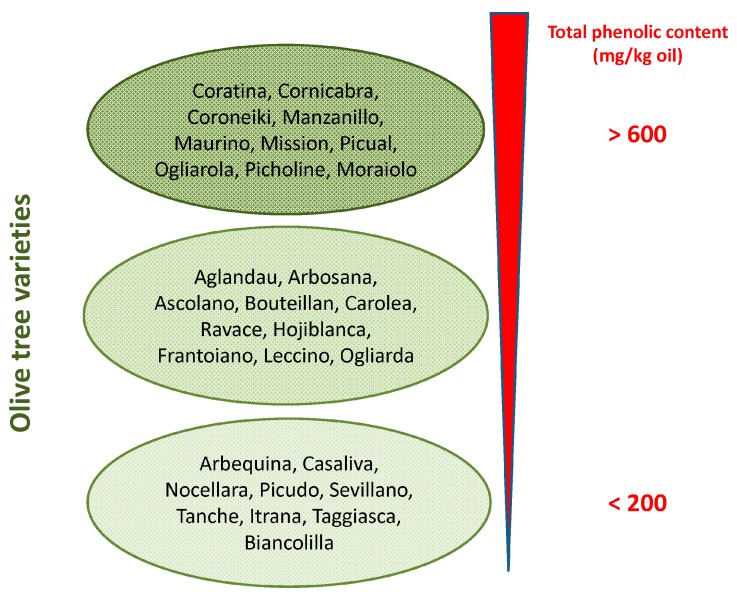
Total polyphenol content in more widespread olive tree varieties.

Phenolic concentration in EVOO depends on several variables such as (i) the olive cultivar (Figure 1) and the ripening stage of fruit [17]; (ii) environmental factors (altitude, cultivation practices, and amount of irrigation); (iii) extraction conditions (heating, added water and malaxation); (iv) extraction systems used to separate oil from olive pastes (pressure, centrifugation systems); and (v) storage conditions and time, due to spontaneous oxidation, and suspended particle deposition [18]. At best, the content of OLE in EVOO can reach levels exceeding 60 mg/100 g (Figure 1).

## 3. Biochemical Effects of Olive Polyphenols Considered as Caloric Restriction Mimickers

The high content in plant polyphenols, one of the main features underlying the beneficial effects of the MD, is provided mainly by the use of EVOO, the main source of alimentary lipids. The wide and increasingly recognized beneficial properties of plant polyphenols have led to proposing them as nutraceuticals and the aliments containing them as functional foods. The latter are defined as “Natural or processed foods that contain known or unknown biologically-active compounds; these foods, in defined, effective, and non-toxic amounts, provide a clinically proven and documented health benefit for the prevention, management, or treatment of chronic diseases” [19]. Extensive research, clinical trials, epidemiological and observational studies have for a long time described the close association between the MD and the Asian diet regarding their polyphenols and a number of physiological and metabolic effects [1,20,21,22,23]; the latter, most often, are similar to those associated with caloric restriction (CR) in humans [24,25], indicating that these substances are CR mimickers [26] (Table 1).

The effectiveness of CR to prolong lifespan and to reduce the risk of age-associated diseases is widely recognized [25]. However, a CR regime can hardly be sustained for long periods of time; this is why diet integration with factors able to mimic the beneficial effects of a reduction of caloric intake can be highly appreciated. Plant polyphenols, including olive ones, induce CR-like effects in muscle, brain, fat tissue and kidney in several ways, particularly through the activation and increased levels of sirtuins (Sirt) [26,27].

Sirt are NAD-dependent type-3 deacetylases [28] whose activity is modulated by the metabolic state of the cells and induced by CR. More contradictory data on CR-induced Sirt1 changes have been reported in liver, where decreased Sirt1 levels were found with an ensuing decrease of hepatic fat synthesis and accumulation [29]. Sirt are involved in lifespan and metabolism regulation in varying organisms [26,29]. Among the Sirt family, Sirt1, the most investigated, protects the cells against oxidative stress and DNA damage. Many of the cellular effects of Sirt1 are mediated by gene regulation following its ability to control the acetylation/deacetylation state, and hence the activity, of several transcription factors, including p53, FOXOs, NFκB, Nrf2, PPARα/γ, PGC1α and LXR [30,31,32,33,34,35]. These factors are known to be involved in the control of apoptosis, autophagy, cell proliferation, oxidative stress, inflammation, protein synthesis, carbohydrate and lipid metabolism. Sirt induction by CR, resveratrol or other plant polyphenols [36] results in many cellular outcomes and is considered responsible for the epigenetic effects of these molecules [5] (see below). In particular, it has been reported that Sirt induction (i) counteracts elevated inflammation and lowers cholesterol and triglyceride synthesis [37]; (ii) reduces oxidative damage markers and increases expression of Nrf2-dependent genes that modulates antioxidant factors in mice fed with a diet rich in olive oil phenolics [38]; (iii) activates Nrf2 thus attenuating oxidative stress with endothelium protection in mice fed with resveratrol [39]; (iv) downregulates the pro-inflammatory agent NFκB thus inhibiting the inflammatory response in rat heart subjected to myocardial ischemia and reperfusion [40]; and (v) inhibits directly the transcriptional activity of PPARγ, with ensuing anti-adipogenic effects [41]. In addition, a cross-talk does exist between Sirt1 and AMP-activated protein kinase (AMPK), a sensor of the energy state of the cell. The AMPK–Sirt1 relation results in mutual activation [42] with modulation of the response of the organism to limited nutrients or increased energy demand and autophagy activation. Actually, the latter, in addition to CR [43], has been reported to be induced by Sirt1 and AMPK [44] that, in turn, can be activated by resveratrol [45], or by olive polyphenols [46,47,48].

The data reviewed above indicate that most of the biochemical and physiological effects of plant polyphenols go well beyond their known antioxidant power and support mechanistically their apparent protection against a number of diseases (see below). In this respect, as pointed out above, EVOO polyphenols, notably OLE, have been shown to directly modulate the insulin/IGF1/AKT and the mTOR pathways, whose downregulation results in FOXO3 activation with ensuing transcription of homeostatic genes favoring longevity and reducing inflammatory states. mTOR, a master regulator of cell life, is one of the most potent upstream regulators of autophagy; activation of the latter appears as one of the ways olive polyphenols can induce most of their beneficial effects against neurodegeneration [46,47]. Genetic inhibition of autophagy results in degenerative modifications in mammalian cells that compromise the longevity-promoting effects of CR and recall the aging-associated ones; conversely, normal or pathological aging is often associated with impaired autophagy [49]. A plethora of studies has clearly shown that plant polyphenols, including those in the olive tree, control the phosphorylation state of signaling molecules such as PI3K, Akt, eNOS, AMPK and STAT3; these are involved in the mechanism of ischemic preconditioning [50] and in autophagy promotion via Sirt1 activation and/or via Ca^2+^ increase with ensuing stimulation of the calcium/calmodulin-dependent protein kinase kinase β (CAMKKβ)-AMPK-mTOR pathway [48]. In cancer cells, the same polyphenols appear to promote cell death by stimulation of apoptosis with features that appear to depend on the cell type [51,52,53], as better specified below.

Overall, the data currently available support the idea that different plant polyphenols, including those from the olive tree, are able to mimic CR effects by affecting the same, or very similar, cellular targets and can therefore be taken into consideration for prevention and/or long-term treatment of aging-associated diseases resulting from chronic inflammation or transcriptional, redox or metabolic derangement.

## 4. Possible Uses of Olive Polyphenols in Disease Prevention and Therapy

**Figure 2:**
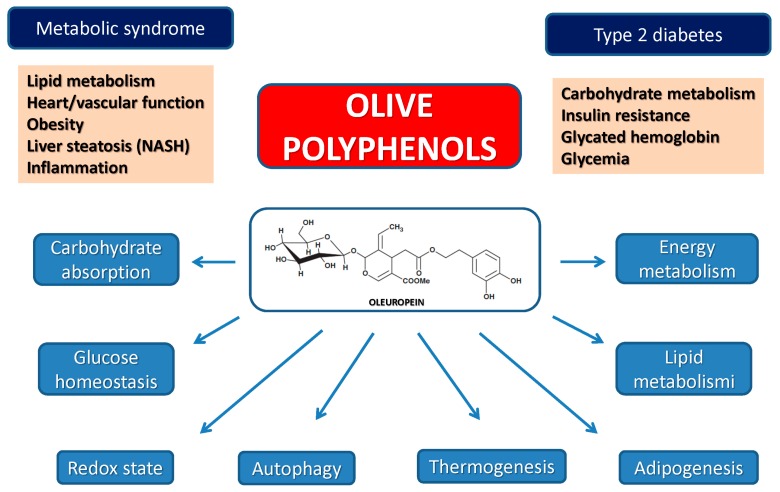
Schematic panel showing the main biochemical effects with their medical significance of oleuropein and olive polyphenols.

The above conclusions are confirmed by an increasing body of studies carried out on cultured cells, model animals and humans (for the latter, see also Section 6). These studies provide compelling evidence that plant polyphenols, including olive polyphenols, are potential candidates for prevention and therapy of a number of diseases and pathological conditions, particularly cancer and several aging-associated degenerative diseases [54] (Figure 2). The following section summarizes recent studies on olive polyphenols providing support to this view.

### 4.1. Olive Polyphenols and Cancer

Information on the anticancer power of olive polyphenols is increasingly available. The data refer mainly to studies carried out on cultured mesothelioma, pancreatic, hepatoma, HeLa, prostate and particularly breast cancer cells, and also on tumors in animal models. These studies have highlighted the effects of OLE and HT on calcium dynamics by acting on ion T-type Ca^2+^ channels leading to increased calcium concentrations and impaired cell proliferation [55,56]. A number of studies have highlighted the anti-proliferative and pro-apoptotic effects of olive polyphenols on cancer cells [57] leading to the conclusion that these effects stem from different mechanisms depending on the cell type (Table 2).

OLE and HT were shown to reduce angiogenesis via downregulation of cyclooxygenase-2 (COX-2) expression, prostanoid production and matrix metallopeptidase 9 (MMP-9) protein release, together with reduction of intracellular ROS levels and NFκB activation [58]. Polyphenol-stimulated apoptosis has been reported to proceed (i) via caspase activation involving pro-apoptotic Bcl-2 family members and PI3K/AKT signaling in pancreatic cancer and hepatoma cells [51,56] and (ii) through the dose-dependent cytoplasmic increase of the c-Jun-N-terminal kinase (cJNK), p53, p21, Bax and cytochrome c in HeLa and cervix carcinoma cells [52]. The activation of the p53 or the G protein-coupled estrogen receptor 1/30 (GPER1/GPR30) pathway [53,59], or the inhibition of the anti-apoptotic and pro-proliferation protein NFκB and cyclin D1, its main oncogenic target, in breast cancer cells [60] has also been shown.

Olive phenols seem to be particularly effective in breast cancer cell models. In these cells, HT was shown to induce cell cycle arrest in the G0/G1 phase by a decrease in the cyclin D1 level [61] and OLE apparently prevents cancer metastasis by increasing the tissue inhibitors of metalloproteinases (TIMPs) and by suppressing the MMP gene expression [62]. OLE has also been reported to inhibit aromatase, a cytochrome P450 family enzyme proposed as an important pharmacological target for breast cancer treatment [63], and to induce a complete recovery of sensitivity to trastuzumab (>1000-fold increase) in SKBR3/Tzb100 cells, a model of acquired resistance. The latter effect provides one of the first examples of how selected nutrients provided by an EVOO-enriched MD (particularly OLE aglycone) affect positively Human epidermal growth factor receptor (HER2)-driven breast cancer [64].

OLE is able to reduce cell proliferation through inhibition of fatty acid synthase (FAS) gene expression in certain colorectal cancer cells [65] and in prostate cancer cells. The latter effect results from reduced cell viability and the induction of both thiol group modifications, of reactive oxygen species (ROS) as well as of the expression of γ-glutamylcysteine synthetase, pAkt and heme oxygenase-1 [66]. OLE has also been reported to disrupt actin filaments with ensuing disassembly of cytoskeleton in different cell lines [67] and to shut down, in MDCK cells, epithelial–mesenchymal differentiation, a key process in the progression toward organ failure and metastasis of organ fibrosis and cancer [68].

A recent review has summarized data concerning the anti-cancer activity of olive polyphenols reported in literature, proposing that the effect of EVOO secoiridoids is related to the activation of gene signatures associated with protection against cell aging and stress, including ER stress and the unfolded protein response, Sirt1 and Nrf2 signaling [69]. Moreover, EVOO polyphenols activate AMPK, promote apoptosis in cancer cells and suppress several genes related to the Warburg effect and to cancer stem cell renewal. Finally, EVOO polyphenols prevent age-related changes in cell size, heterogeneity, arrangement and staining of human fibroblasts for β-galactosidase, associated with cell senescence, at the end of their proliferative life period [69]. As such, EVOO polyphenols can be considered a new family of plant-produced gerosuppressants that molecularly “repair” the AMPK/mTOR-driven path, leading to prevention against aging and age-related diseases, including cancer [69].

In conclusion, the data presently available support the idea that OLE and other olive polyphenols hold promise as potential chemotherapeutic agents for treatment of malignant mesothelioma, breast, pancreatic, prostate cancer and other types of tumors. Nevertheless, these potential beneficial effects need full confirmation in animal models and extensive investigation in human subjects before they can be proposed as new possible anti-cancer agents.

### 4.2. Olive Polyphenols and Cardiovascular Disease (CVD)

Cardiovascular endothelium is a main target of all major risk factors for heart disease (hypertension, hyperglycemia, hyperlipidemia, inflammation, aging), and its damage is one of the first steps in the development of CVD. Cardiomyopathy can also be caused by a number of drugs, including the antineoplastic antibiotic doxorubicin [70]. The key feature shared by the CVD risk factors is increased ROS; in turn, ROS production by endothelium mitochondria contributes significantly to heart disease [71,72]. Olive polyphenols (OLE, HT, oleacein, elenolic acid and thyrosol) display important beneficial properties against atherosclerosis [73] and CVD (reviewed in [74]) (Table 3). Finally, at nutraceutical doses, OLE was reported to protect against doxorubicin-induced cardiomyopathy in rat models by positive modulation of oxidative stress markers [75], AMPK activation and iNOS suppression [76]. The strong antioxidant properties of these substances, particularly HT and OLE [74], could explain, at least in part, these effects. In fact, OLE, HT and tyrosol are able to reduce the kinetics and the extent of lipid peroxidation [77] and protect against ischemia/reperfusion-induced oxidative stress provided to isolated rat hearts at doses corresponding to the average intake in a normal MD [78]. Moreover, OLE has been reported to reduce the extent of infarcted tissue, total cholesterol and triglyceride levels in both normal and hypercholesterolemic rabbits, thus providing cardioprotection even before the ischemic event [79]. Taken together, these data warrant further studies to provide convincing support of the future use of olive polyphenols, or some derivatives, as cardioprotective agents.

Olive oil, and particularly its oil polar lipid extract, have been shown to be antiatherogenic by reducing the level of platelet-activating factor, thus reducing platelet aggregation [80]. OLE appears to possess the highest anti-atherosclerotic power among all olive polyphenols, mostly resulting from cholesterol regulation. In particular, EVOO polyphenols have been shown to enhance the expression of genes related to cholesterol efflux from cells to HDL in humans [81] and also to promote the HDL-dependent cholesterol efflux by increasing HDL size, stability and resistance against oxidation [82]. The anti-atherosclerotic effects of OLE in atherosclerotic rabbits has also been reported to imply a reduction of serum levels of total cholesterol, LDL, HDL, triglycerides, NFκB, and several chemokines [83]. In this sense, a potent anti-atherosclerotic power was also reported for thyrosol through activation of a molecular pathway starting with protein kinase B (PKB)/AKT phosphorylation, Sirt1 expression and deactivation of the transcription factor FOXO3, which upregulates pro-apoptotic genes and eNOS [84]. These data were confirmed by subsequent studies showing that olive oil polyphenols downregulate pro-atherogenic genes in healthy humans in the context of a traditional MD [85] and positively modulate post-prandial vascular function (arterial stiffness) and the inflammatory status (interleukin-8, IL8, production), two factors of CVD risk *in vivo* [86]. The latter effects were elicited by repressing the expression of several pro-inflammatory genes, including those encoding the cytokines IL-6 and IL-8 [87], possibly through their redox activity. It must be noted that IL-8, and other pro-inflammatory cytokines, have been proposed to play an important role in the development of atherosclerosis [88], and its circulating levels have been associated with future risk of CVD [89]. Finally, cardioprotection by olive polyphenols has been supported by studies showing their ability to enhance fat oxidation and to optimize cardiac energy metabolism in high-fat-diet rats as well as to improve myocardial oxidative stress in standard-fed rats [90].

Over the course of the last decade, in addition to the already quoted effects on the classical risk factors for CVD, several scientists have studied the beneficial effects of olive oil and olive leaf extracts on thrombosis-associated factors (primary and secondary hemostasis, platelet aggregation, fibrinolysis), a pathophysiological condition closely related to CVD [91,92]. Finally, pre-treatment with OLE and oleacein of endothelial progenitor cells that produce neovascularization of ischemic tissue and *de novo* formation of endothelium in injured arterial walls, increased cell survival and reduced senescent cells and intracellular ROS production, possibly following activation of the Nrf2/heme oxygenase pathway [93].

Overall, these and other data support the notion that olive phenolics can be beneficial for cardiovascular health, suggesting their importance to lower the risk of CVD.

### 4.3. Olive Polyphenols, Obesity, Type 2 Diabetes and the Metabolic Syndrome

EVOO polyphenols display a multifaceted activity against metabolic disorders (Table 4). Obesity, an increasingly widespread condition affecting millions of people mainly in developed countries, is also associated, among others, with increased risk of CVD. Several olive leaf components, including OLE, HT and others have been shown to be effective against obesity by suppressing dose-dependently intracellular triglyceride accumulation and the expression of adipogenesis-stimulating factors during adipocyte differentiation [94,95,96]. Other studies have reported that olive polyphenols can be effective in reducing food intake and fat tissue accumulation by regulating the expression of molecules involved in adipocyte proliferation and thermogenesis at the mitochondrial level [97]. Finally, it must be reminded that most of these effects are shared with other plant polyphenols such as resveratrol, epigallocathechin and curcumin. The latter have been reported to reduce body weight, fat mass and triglycerides by lowering adipocyte viability and preadipocyte proliferation (i) by suppressing adipocyte differentiation and triglyceride accumulation; (ii) by stimulating lipolysis and fatty acid oxidation and (iii) by reducing obesity-associated inflammation [98].

**Figure 3:**
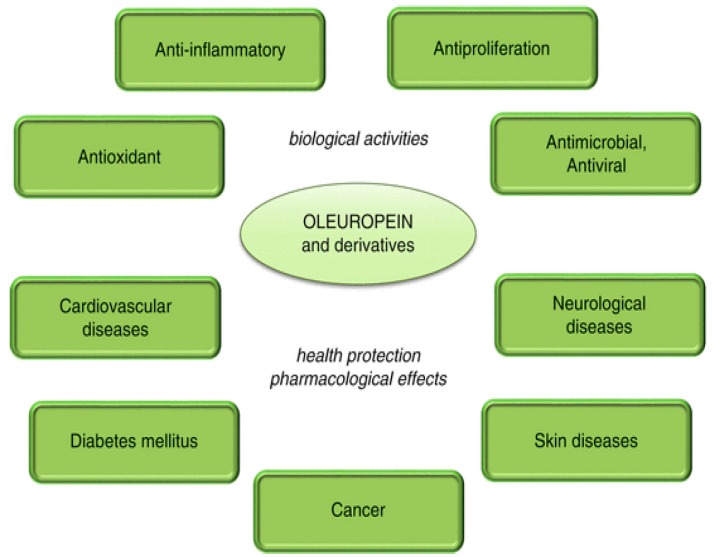
Schematic panel showing the main molecular effects of olive polyphenols counteracting type 2 diabetes and the metabolic syndrome.

Diabetes, notably type 2 diabetes (T2DM), is a condition closely associated with obesity and CVD and these states concur with the so-called metabolic syndrome; the latter also includes non-alcoholic fatty liver disease (NAFLD), a condition whose severity spans from simple triglyceride accumulation in the liver parenchyma (steatosis) to non-alcoholic steatohepatitis (NASH). T2DM and other pathological states associated with deregulation of carbohydrate and lipid metabolism can be positively targeted by olive oil polyphenols. The positive outcome of the administration of OLE and other olive polyphenols against derangement of carbohydrate metabolism is supported by many studies. The latest reports have highlighted that the molecular determinants of these effects are interwoven with those associated with the reduction of obesity and liver steatosis as well as with cardioprotection (see above) (Figure 3).

The reported anti-diabetic effects comprise: (i) the inhibition of the amylin tendency to aggregate into amyloid, whose toxic deposits in the pancreatic β-cells are considered to affect cell viability in T2DM [99]; (ii) the reduction of serum glucose and cholesterol levels with restoration of the antioxidant perturbations in rat [100,101] and rabbit [102] models of diabetes; (iii) the modification of the expression of genes implicated, among others, in lipogenesis, thermogenesis and insulin resistance in high-fat-diet mice [97]; (iv) the reduction of the digestion and intestinal absorption of dietary carbohydrates both in the mucosal and in serosal sides of the intestine of diabetic rats [101], together with the improvement of glucose homeostasis with a reduction of glycated hemoglobin and fasting plasma insulin levels in humans [103,104]; (v) a significant rise in insulin sensitivity and pancreatic β-cell secretory capacity in middle-aged overweight men [104] with a measurable and rapid change of the expression of genes mechanistically related to insulin sensitivity and to the metabolic syndrome [105]; (vi) the reduction of the metabolic activity, cardioprotection and prevention of inflammation and of cytokine-induced oxidative damage of pancreatic β-cells [98,106]; (vii) the improvement of the antioxidant status in healthy elderly people [107] and the protection of insulin-secreting β-cells against H_2_O_2_ toxicity by modulating redox homeostasis with protection of cell physiology against oxidative stress [108]; (viii) the prevention of the inflammatory response and of cytokine-mediated oxidative cell damage with downregulation of the genes involved in adipocyte differentiation [93]; (ix) the downregulation of Wnt10b inhibitory genes and upregulation of β-catenin protein expression as well as of key adipogenic and thermogenic genes in high-fat-diet mice [95,109] as well as of genes involved in galanin-mediated signaling; (x) the upregulation of genes involved in Wnt10b-signaling in C57BL/6N mice [110] and (xi) the increase of signal molecules active in fasting conditions (IL-6, Insulin-like growth factor-binding protein 1 and 2, IGFBP-1 and IGFBP-2), yet in the absence of significant effects on interleukin-8, TNF-α, CRP, lipid profile, liver function ambulatory blood pressure and thickness of carotid wall layers [102].

Finally, two recent studies have reported that (i) the anti-oxidant and anti-inflammatory properties of the polyphenols in olive leaf extracts attenuate the metabolic, structural and functional modifications in the heart and liver of rats with diet-induced metabolic syndrome [111] and (ii) the administration of an OLE-enriched supplement to the Tsumura, Suzuki obese diabetes (TSOD) mouse model of T2DM reduced hyperglycemia and impaired glucose tolerance and, less evidently, oxidative stress, when the administration was extended in the long term. In this study, OLE supplementation was ineffective in reducing obesity [112]. In other cases, the anti-obesity and anti-steatosis effects of olive polyphenols have been associated with increased lipid metabolism and energy expenditure as well as with the modulation of glucose homeostasis mentioned above [96].

Olive polyphenols display significant protection against liver disease resulting from altered lipid metabolism. In particular, OLE protects HepG2 and FL83B cells against free fatty acid (FFA)-induced hepatocellular steatosis via reduction of FFA-induced lipogenesis following lowered extracellular-regulated kinase (ERK) activation, reduced expression of genes involved in adipocyte differentiation, and Wnt10b inhibition in hepatocytes [113]. OLE also appears to protect against oxidative stress-mediated liver damage by increasing the expression of genes involved in liver lipogenesis, oxidative stress and inflammatory response [114] as well as by reversing, in visceral adipose tissue, the downregulation of thermogenic genes involved in uncoupled respiration and mitochondrial biogenesis induced by a high fat diet [95]. Moreover, olive polyphenols have been reported to downregulate lipid synthesis in primary cultured rat hepatocytes through AMPK phosphorylation, suggesting that a decrease in hepatic lipid metabolism, particularly lipid synthesis, may represent a possible mechanism underlying the reported hypolipidemic effect of these substances [115,116]. Olive oil and polyphenols, notably OLE, also prevent NAFLD (reviewed in [117]) and its progression to NASH and liver fibrosis in mouse models of NASH, presumably through anti-oxidant activity and reduced lipid accumulation, supporting their potential pharmacological use in NASH prevention and care [118,119,120]. Finally, other studies carried out in animal models and with cultured cells have shown that one of the mechanisms of cell protection by OLE is the aforementioned potent stimulus to autophagy. Presently, autophagy stimulation is considered an important potential goal in the research of effective therapies against neurodegenerative and dysmetabolic pathologies such as T2DM.

### 4.4. Olive Polyphenols and Amyloid Diseases

Amyloid diseases provide examples of the way plant polyphenols may interfere with specific pathologies (reviewed in [121]). Amyloidoses are a number of sporadic or familial degenerative conditions characterized by misfolding and aggregation into intractable polymeric fibrillar assemblies of a number of specific peptides/proteins (reviewed in [122]). These aggregates are found as intra- or extracellular deposits that are currently considered among the main factors affecting cell physiology and viability. Amyloid diseases comprise rare pathologies but also largely diffused diseases such as T2DM, AD and PD [123]. Many plant polyphenols, and among them those found in EVOO (Table 5), have been shown to interfere in different ways with the aggregation path, reducing aggregate load and its cytotoxic effects [123].

The observation that patients with diabetes have an increased risk of developing AD compared to healthy individuals has recently led to the proposal that AD may be associated with brain insulin resistance. Actually, many studies have shown that insulin resistance, increased inflammation and impaired metabolism are key pathological features of both AD and diabetes (reviewed in [124,125]). Emerging evidence underscores the importance for AD development of brain insulin resistance, a key alteration in pre-diabetes and diabetes mellitus. Such a relation has led some authors to propose some AD symptoms as a type 3 brain diabetes (reviewed in [126]). Actually, insulin and insulin-like growth factors appear to regulate several biological processes at the basis of learning and memory, including energy metabolism, synaptic plasticity and axonal growth. It was also reported that a hyperinsulinemia-induced condition of insulin resistance results in the activation of glycogen synthase kinase 3β, a key factor for cognitive decline, with ensuing brain injury. Hence, the endogenous impairment of insulin signaling in the brain accounts for important AD abnormalities.

Other factors link diabetes to neurodegeneration, including a shared role of amylin found aggregated both in the AD brain and in T2DM pancreatic β-cells [127]. OLE and oleocanthal have been shown to interfere with the amyloid aggregation of Aβ, amylin and tau. The latter is a microtubule-associated protein found aggregated in several tauopathies, including AD. The data reported indicate that OLE [128,129] and oleocanthal [130,131] interfere with the aggregation path of these peptides/proteins upon binding to the aggregating molecules, skipping the appearance of toxic species and favoring the formation of non-toxic disordered aggregates. Interestingly, the use of the aggregating Aβ peptide has allowed to distinguish two different mechanisms by which polyphenols and their glycosides interfere with the amyloid aggregation of this peptide. In fact, amyloid oligomers are remodeled by the aglycones by rapid conversion into large off-pathway aggregates, whereas they are rapidly dissociated into soluble disaggregated peptide molecules by the glycones [132]. The binding site of OLE to Aβ has also been described [133]. OLE also activates autophagy and reduces the inflammatory response provided by the accumulation of amyloid aggregates of Aβ and its pyroglutamylated 3–42 derivative in the affected brain areas of mouse models. As a result, the administration of OLE aglycone to TgCRND8 mice, a transgenic model of Aβ deposition, results in reduced plaque load and astrocyte reaction, with strong improvement of memory and behavioral performance to the levels recorded in wild-type mice, with respect to untreated littermates, [47,48]. These data confirm previous findings obtained with an Aβ peptide-expressing transgenic model of *C. elegans* displaying Aβ aggregates in the skeletal apparatus that disappeared in the OLE-fed worms [134]. Finally, other studies indicate that oleocanthal enhances amyloid-β clearance from the brain [135] and that OLE reduces Aβ production by promoting the non-amyloidogenic pathway through increased α-secretase cleavage of the amyloid precursor protein [136].

Taken together, these data provide molecular and biological insights strongly supporting the protection by olive polyphenols against age- and lifestyle-associated neurodegeneration (reviewed in [137]) even though data on protection by olive polyphenols against neurodegeneration in humans are lacking.

## 5. Epigenetic Effects

Epigenetics is defined as the complex of heritable changes to the transcriptome that are distinct from those resulting from the base sequence in the genome but are associated with post-transcriptional gene regulation by non-coding RNAs and with histone and DNA chemical modifications; the latter include DNA methylation, histone methylation, acetylation and phosphorylation [138]. Non-coding RNAs include microRNAs (miRNAs), small non-coding RNAs that post-transcriptionally modulate gene expression. miRNAs contribute to the control of the expression of both DNA methyltransferases (DNMTs) and histone-modifying enzymes and influence many cellular processes including survival of neuronal cells (reviewed in [139]). Epigenetic modifications regulate gene expression in a synergistic and cooperative way by changing chromatin arrangement and DNA openness, thus switching on/off a number of genes associated with important physiological and pathological processes (aging, and age-related pathologies, including cancer and neurodegeneration, reviewed in [140]). These changes are acquired throughout life, including embryonic and fetal development, and depend on environmental clues such as diet, lifestyle and exposure to toxins. When epigenetic modifications are inherited following cell division, they result in the enduring maintenance of the acquired phenotype; however, they can also occur at some time in the course of life and, as such, may remarkably influence phenotypic outcomes in terms of health, disease or risk of disease [5].

Although numerous compounds have been developed to specifically alter the function of chromatin-modifying enzymes, for example, histone deacetylase (HDAC) inhibitors, we are only beginning to understand the epigenetic effects of dietary compounds. Well-known examples of dietary chromatin-modifying compounds include curcumin, the active constituent of turmeric, which has been shown to be an HDAC inhibitor, as well as the red wine phenol, resveratrol, which activates Sirt1 [141,142]. Actually, the investigation of epigenome modifications following administration of plant polyphenols dates back less than one decade. Since then, an increasing number of studies has clearly shown that plant polyphenols, as other nutrients, directly regulate both transcriptional and translational processes by modulating the activity and expression levels of enzymes involved in chemical modifications of histones and DNA (reviewed in [5]). A growing body of evidence suggests that epigenetic changes triggered by diet nutrients, including plant polyphenols, contribute to preventing some diseases, notably cancer. In particular, plant polyphenols can counteract aging as well as many of its pathological consequences resulting from aberrant epigenetic mechanisms [52,139,143,144,145]. In the context of neurodegenerative diseases, the epigenetic modifications have been shown to induce effects similar to those provided by CR [146]. Accordingly, epigenetics issues targeted by diet polyphenols have become an attractive approach for disease prevention and intervention and provide a rationale for most of the anti-cancer and anti-neurodegeneration power shown by these substances. Epigenetic therapy is an expanding field and is providing clues useful for discovering new drugs, some of which are undergoing clinical investigations, mostly as anti-cancer drugs [147,148]. The latter include plant polyphenols such as genistein and quercetin, exploited for their activity as HDAC and DNMT modulators.

The studies reported in the last decade have been carried out mostly on cancer cells and the modifications of gene and protein expression profiles underlying many of the effects listed above can be ascribed to recently shown epigenetic modifications elicited by many plant polyphenols; these include resveratrol, curcumin, epigallocathechins, genistein, quercetin and others in the anti-diabetic, anti-ageing and anti-neurodegeneration fields [140]. Information about the role of olive polyphenols as epigenome modulators is quite scarce as opposed to other polyphenols. Recent data show that OLE aglycone given orally for eight weeks to TgCRND8 mice, a model of Aβ deposition, downregulates HDAC2 [48], an enzyme known to be upregulated in AD [149]. In these mice, the downregulation of HDAC2 resulted in a significant increase in the level of histone acetylation, in particular of H3 at K9 and of H4 at K5 [48]. Histone acetylation has been reported to improve cognitive deficits in animal models of AD and its indication is considered a promising novel therapeutic strategy against AD [150]. A recent *in silico* molecular modeling study combined with known experimental affinities for controls has identified potential chromatin-modifying compounds from *Olea europaea*; in particular, HT was highlighted as a potential inhibitor of HDAC6 and lysine-specific histone demethylase 1 (LSD1) following its high affinity for binding to the active site of various chromatin-modifying enzymes [151]. Other recent studies have shown that an olive oil-enriched diet increases global DNA methylation in the mammary gland and in a murine model of breast cancer induced by dimethylbenz(a)anthracene (DMBA) [152]. Finally, EVOO or its phenolic compounds have been reported to modulate the expression of the CNR1 gene encoding for the type 1 cannabinoid receptor via epigenetic mechanisms, both in rats and in human Caco-2 colon cancer cells [153].

In spite of the limited information on the epigenetic effects of olive polyphenols presently available, the similarities between many effects of different plant polyphenols at the molecular level hold promise that the well documented epigenetic effects reported for many other plant polyphenols could be largely retrieved also in olive polyphenols. In conclusion, modulation of epigenetic flaws by natural polyphenols appears as a promising subject for the discovery of new compounds effective against chronic diseases, even though the present clinical studies have been dedicated prevalently to deciphering polyphenol effects in cancer treatment. HDACs and DNMTs are promising objectives for the control of human pathologies; the modulation of their activity is affected by plant polyphenols, some of which are found in significant amounts in widely used foods that characterize the MD and the Asian diet. However, the data on the epigenetic effects of olive polyphenols are still scarce and further research is needed to increase the information necessary to propose the possible use of these substances as epigenome modulators in humans.

## 6. Epidemiological Studies and Clinical Trials with Olive Oil and Its Polyphenols

During the last decade, the beneficial properties of EVOO and EVOO polyphenols for human health have been assessed in epidemiological studies and clinical trials. A number of these studies, often carried out on a limited number of patients, have been cited in the preceding sections [83,84,87,103,104,107]. In this section, the studies carried out on large cohorts of participants will be reviewed (Table 6).

One of the most cited surveys on protection against neurodegeneration and cognitive decline by olive oil is the “Three-City Study” that enrolled around 7000 elderly subjects. The results showed lower odds of cognitive deficit for those subjects who used olive oil moderately (just for cooking or dressing) or intensively (for cooking and dressing) as opposed to those who never used it [154]. In the same cohort, a lower incidence of stroke in people using higher amounts of olive oil was also observed [155]. The results of this study were confirmed by two multicenter, randomized, controlled trials, the so-called PREvención con DIeta MEDiterránea (PREDIMED) and PREDIMED-NAVARRA studies carried out in Spain on people at high cardiovascular risk. The PREDIMED study was a primary prevention trial originally designed to test the long-term effects of the MD on the incidence of CVD in people with high cardiovascular risk. The cohort was also evaluated for cognitive performance, after adjustment for several potentially interfering factors. It emerged that an intervention with a MD enriched in EVOO (better than with wine or nuts) significantly improved cognition, and that EVOO phenolic content was the main factor responsible for this result [21,156]. The main goal of the study was assessing CVD prevention; in this sense, the trial suggested that EVOO consumption is associated with a reduced risk of CVD and mortality [157,158].

The role played by polyphenols in cardiovascular protection was further confirmed by two sub-studies on patients subjected to MD high in polyphenols (from nuts or EVOO). Total polyphenol excretion by people adopting a MD rich either in nuts or EVOO was positively correlated with changes in plasma levels of triglycerides, glucose and nitric oxide (NO); moreover, the statistically significant increase in plasma NO levels was associated with a reduction in systolic and diastolic blood pressure [159]. Accordingly, data from a subsample (*n* = 990) of the PREDIMED study have shown that the MD significantly decreases LDL oxidation only when it is enriched in EVOO with medium-high phenolic content [160].

Overall, as outlined by a recent survey, the PREDIMED study in patients at the MD rich in phenols (from nuts or EVOO) showed a significant improvement of classical and emerging CVD risk factors, including inflammation, oxidative stress blood pressure, carotid atherosclerosis, insulin resistance, lipoproteins and lipid profiles [161]. Breast cancer incidence was also investigated in the PREDIMED cohort. Thirty-five out of the 4282 women enrolled in the survey displayed confirmed cases of invasive breast cancer, whose risk was reduced by 68% in the EVOO group as compared with the low-fat group even after accounting for factors such as age, body mass index, exercise and drinking habits. The risk of being affected by invasive breast cancer was highest for women who were instructed to eat less fat (2.9 cases for 1000 person-years). This value was compared to a diagnosis rate of 1.8 cases per 1000 person-years for women on the MD supplemented with nuts and a rate of 1.1 cases per 1000 person-years for women on the MD with increased EVOO, suggesting a better protection against breast cancer of EVOO over nuts [162].

More recently, a cross-sectional sampling population study was carried out among the whole Spanish people. The study selected, in 100 health centers, 4572 individuals aged >18 years, representative of the Spanish population. Clinical, demographic and lifestyle parameters were considered, together with physiological parameters (body weight and height, body mass index, waist and hip measurement, blood pressure and oral glucose tolerance). The participants were analyzed considering whether they were consumers of olive oil or sunflower oil. The main outcome of the study showed that the consumption of olive oil was associated with significant beneficial effects on several cardiovascular risk factors, particularly in the presence of obesity and a sedentary lifestyle, and with a significant improvement of impaired glucose tolerance and insulin resistance [163]. Another study aimed at examining the association between olive oil intake and the incidence of T2DM was carried out in the USA by following 59,930 women aged 35–65 years from the Nurses’ Health Study (NHS) and 85,157 women aged 26–45 years from the NHS II with no diabetes, CVD and cancer at baseline. The diet was controlled and validated by food-frequency questionnaires. Hidden T2DM cases were identified and confirmed by questionnaires. At the end of the 22 years follow-up, 5738 and 3914 cases of T2DM were documented in the NHS and NHSII, respectively. The outcome compared people taking at least one tablespoon of olive oil daily with those who never assumed olive oil. The results suggested that higher olive oil intake was associated with a modestly lower risk of T2DM; conversely the risk was raised in people who substituted olive oil with other lipids [164]. Finally, a very recent study has been conducted with 25 healthy subjects randomly allocated in a cross-over design to a Mediterranean-type meal supplemented with or without 10 g EVOO/day or the same amount of corn oil. The lipid profile and glycemic parameters related to glucose tolerance, determined two hours after the meal, showed that EVOO improves post-prandial glucose and LDL-cholesterol, confirming the anti-atherosclerotic power of the MD [165].

Overall, these surveys support the notion that natural EVOO phenols might counteract age-associated cognitive decline, CVD and cancer, particularly breast cancer. Accordingly, many clinical trials have been conducted employing olive oil or polyphenol-enriched olive extracts, but the results are still scarce even from those studies that were completed, except for trials aimed at investigating the effect of EVOO and its polyphenols against several conditions associated with CVD (oxidative stress, inflammation, haemostasis, endothelial function and blood pressure). In a small crossover trial, carried out in the context of the EUROLIVE (Effect of Olive Oil Consumption on Oxidative Damage in European Populations) study (Trial number: ISRCTN09220811), 200 participants were subjected to three rounds of daily administration of 25 mL of three olive oils with a different phenolic content (low, 2.7 mg/kg of olive oil; medium, 164 mg/kg; medium-high, 366 mg/kg) for three weeks preceded by a two-week washout period. The results showed a linear decrease of the total cholesterol/HDL-cholesterol ratio and of oxidative stress markers with the increase of the phenolic content of the olive oil [166]. Protection against atherosclerosis was confirmed by a sub-set of the same trial with 25 healthy volunteers fed for three weeks with 25 mL/d uncooked olive oil with a medium-high polyphenol-content (366 mg/kg) or a low-polyphenol-content (2.7 mg/kg) [167]. The results provided a first-level evidence that the phenolic content is the main factor responsible for the health benefits of EVOO. This correlates also with the results coming from the randomised, controlled clinical trial involving pre-hypertensive patients fed with 30 mL of two similar olive oils, a functional EVOO enriched with its phenolic compounds (961 mg/kg,) or a medium polyphenol content EVOO (289 mg/kg). Data from this study support a significant upregulation of genes regulating the cell-HDL cholesterol efflux [81]. That olive polyphenols increase human HDL functionality has recently been confirmed by a subsample of the EUROLIVE study, in which it was shown that the consumption of olive oil with high phenolic content increased the cholesterol efflux from macrophages mediated by HDL [82].

Most of the above studies were aimed at assessing the effects of EVOO and its polyphenols after a relatively long-term ingestion; studies were also carried out to determine the effects of an acute administration of olive oil, particularly in the case of protection against postprandial hyperlipidaemia and the associated inflammation. A study conducted on 20 obese subjects that were given muffins made with different oils previously subjected to 20 heating cycles, showed that oils rich in phenols, whether natural (EVOO) or artificially added, reduced postprandial inflammation; this outcome was determined by the activation of nuclear NFκB, the cytosolic levels of IκB-α, an NFκB inhibitor, the mRNA levels of p65, IKKβ, and IKKα (NFκB subunits and activators) and by the levels of lipopolysaccharide (LPS) and other pro-inflammatory molecules (TNF-α, IL-1β, IL-6, migration inhibiting factor (MIF), JNK); seed oil (sunflower) failed to produce similar results [168]. The protection against post-prandial oxidative stress was confirmed by other randomised, cross-over, controlled human studies showing that the serum antioxidant capacity was increased after EVOO ingestion at the same single doses (40 or 50 mL) at which oxidative stress normally occurs if the ingested oil is not EVOO [169]; the study also reported a lower lipid oxidative damage in subjects fed with an olive oil with high, rather than low, phenolic content [170,171]. Finally, a comprehensive review synthetically reports the results of randomized, controlled trials showing the efficacy of EVOO in lowering numerous inflammation markers such as thromboxan2 (TBX_2_), leukotriene B4 (LTB_4_), intercellular adhesion molecule 1 (ICAM-1) and vascular cell adhesion molecule 1 (VCAM-1), TNF-α, IL-1β, IL-6, MIF, JNK and LPS, NFκB and its activators, high-sensitivity C-reactive protein (hs-CRP), asymmetric dimethylarginine (ADMA) relevant in the context of several pathologies including CVD [172].

## 7. Bioavailability of Olive Polyphenols

A problem associated with the use of olive and other plant polyphenols is their reduced bioavailability due both to incomplete intestinal absorption and to rapid biotransformation favoring urinary excretion. Moreover, in the case of the brain, orally ingested polyphenols must cross an additional barrier, the blood–brain barrier, in addition to that represented by the enterocytes. With few exceptions, only polyphenol aglycones can be absorbed in the small intestine [173] and deglycosylation by β-glucosidase in small intestinal epithelial cells has been focused on as a crucial step in absorption and ensuing metabolism of dietary polyphenols, notably the glycated forms [174]. Once released from the enterocyte into the lymph and therefrom into the blood stream, most polyphenols undergo substantial biotransformations including methylation, glucuronidation, sulphation and thiol conjugation [175] that alter their chemical properties, favor their excretion and, possibly, provide them new biological activities [176]. Moreover, recent research has highlighted the importance for polyphenol bioavailability of the colonic microflora that can extensively metabolize and chemically modify polyphenols [177]. However, recent studies on this theme carried out both on rats and humans have shown that these compounds are indeed absorbed in discrete amounts from the intestine and rapidly distributed through the blood flow to the whole organism, including the brain. In particular, recent data clearly indicate that, similarly to other polyphenols, the glycated and (preferentially) the aglycone forms of OLE are indeed absorbed and found in the plasma after ingestion both in rats [178,179] and in humans [179,180,181,182,183]. From the plasma they, at least in part, are distributed to different organs and tissues, including the brain, where they, or some derivatives, have been found [179]. Finally, a recent metabolite-profiling study with cultured breast cancer cells treated with an olive leaf extract has shown that OLE is the main polyphenol found inside the cells, suggesting its ability to cross the plasma membrane in this cell line [184]. As a confirmation, a very recent study has shown that OLE interacts with synthetic phospholipid membranes, but the extent of the interaction depends on membrane lipid composition and is favored by the presence of anionic lipids, suggesting specific interactions [185]. These data agree with those reported in a recent study showing that a number of polyphenols (those from the olive tree were not included) are able to protect the mitochondrial membrane from permeabilization by amyloid oligomers, suggesting some interference with the formation of the oligomer–membrane complex [186]. Finally, in a recent study we have shown that the OLE metabolite, HT, arising mainly from acid hydrolysis in the stomach, is found in the brain of TgCRND8 mice fed with OLE for eight weeks [48]; this finding agrees with previous data reported in rats [178] supporting the ability of some OLE derivatives, including HT, to cross the blood–brain barrier, even though its generation from OLE once it has crossed the blood-brain-barrier cannot be excluded.

Accurate studies on the effective daily dose of olive polyphenols to be administered to humans to get significant protection are still lacking. What must be taken into account is that, apparently, the amount of OLE and other plant polyphenols present in foods is not adequate to ensure daily doses suitable to get short-term acute effects. However, clinical and experimental evidence suggests that the continuous assumption of foods containing moderate amounts of these molecules can be effective in the long term, also due to their possible accumulation as lipophilic molecules, producing a low-intensity continuative stimulus of cell defenses against oxidative stress, amyloid deposition and other alterations underlying age-associated pathologies. Nevertheless, the low daily consumption of olive oil polyphenols with a typical MD suggests the value of the integration of polyphenol-enriched olive leaf extracts that can intensify, in the short-term, the beneficial effects of these molecules.

## 8. Conclusions

At the end of our itinerary through the nutraceutical properties of EVOO and its polyphenols we should recognize that the research in this field is actively proliferating, hence it is becoming mandatory to accommodate the plethora of biochemical, cellular and physiological effects of EVOO polyphenols in a coherent picture. In fact, looking at the multitude of cellular effects elicited by these compounds, that often look similar to those elicited by other plant polyphenols, one could have the uncomfortable sensation that their action is rather non-specific. Moreover, in some cases, contradictory results have been reported; these can possibly result, among others, from differences in the study design and administered doses of olive polyphenols in the case of clinical trials, the type of animal (race, model, wild-type or transgenic) in the case of animal studies, or the type of investigated cells and cell culture conditions. However, we do believe that some common traits are emerging that will pave the way to mechanistically defining the nutraceutical activity of EVOO and olive polyphenols. These traits can be summarized as follows.

First, plant, notably olive, polyphenols act mainly as signaling molecules. Although the direct target of interaction must still be identified, a calcium-mediated activation of AMPK via CaMKKβ has been reported for OLE aglycone [46]; oleocanthal was also found to activate AMPK [187]. Aging can be considered as a phenomenon driven by over-activation of the nutrient-sensor mTOR gerogene following a decline, or lack of responsiveness to activation signals of AMPK, an energy-sensing protein and a critical mTOR gerosuppressor [69]. AMPK is a sort of node in the intricate signaling network of the cell, where several physiological and pathological pathways (autophagy, anabolism/catabolism, apoptosis, cell proliferation, inflammation, neurodegeneration) intersect [188,189,190,191,192]. Accordingly, AMPK activation by olive polyphenols is a possible explanation for most of the pleiotropic activities of these substances. In general, specific receptor–polyphenol interactions have not been clearly identified; rather, it is believed that these compounds interact freely with the cell membrane bilayer [185,186], modifying its permeability properties, notably to calcium [46]. However, a receptor, the TRPA1 receptor ion channel, spatially restricted to the throat, has recently been identified as the possible responsible for the pungency of oleocanthal sensed in the throat [193].

Second, EVOO polyphenols directly participate to the redox balance of the cell. They perform this role not simply as antioxidants but, in certain circumstances, also as mild pro-oxidants, by up-regulating the antioxidant defenses of the cell, thus acting as hormetic factors [69]. This has been shown for both HT that, in the presence of peroxidases, can undergo a redox cycling that generates superoxide, an inducer of Mn-SOD expression [194] and for tyrosol, that increases *C. elegans* lifespan also by activating the heat shock response [195]. The role of EVOO phenols as “xenohormetic agents” has been analyzed by Menendez and coll. in their transcriptome analysis of cells exposed to crude EVOO polyphenol extracts highly enriched in secoiridoids OLE and decarboxymethyl OLE; their data confirm an involvement, among others, of the activation of anti-aging/cellular stress-like genes, including those for ER stress, the unfolded protein response, Sirt1 and Nrf2 signaling [69].

Third, it is generally believed that plant polyphenols can directly interact with other molecules or molecular complexes through aromatic stacking, hydrophobic interaction, or chemical crosslinking. Actually, this mechanism seems to underlie the inhibition of toxic amyloid aggregation by EVOO phenols. In the case of oleocanthal, it usually promotes the conversion of monomers and oligomers of amyloidogenic proteins/peptides (such as Tau and Aβ) to high molecular weight aggregates by chemical crosslinking thanks to its couple of aldehyde groups [136,196,197]. OLE aglycone differs from oleocanthal by the absence of aldehyde groups and the presence of a methoxycarbonyl group. Therefore, OLE is not a chemical crosslinker and its anti-amyloidogenic activity should occur differently [130]. Structure comparison analysis suggests that the number of phenolic rings is a key responsible for the polyphenol’s efficacy in remodeling amyloid oligomers; in particular, two aromatic rings, at least one with a hydroxyl group, appears to be the minimal structural requirement needed by phenolic aglycones to remodel Aβ oligomers by interfering with aromatic group stacking [132]. Actually, the main EVOO polyphenols do not respond to this description, since they have just one phenolic ring. Nonetheless, it has been clearly shown that OLE physically interacts with the Aβ peptide [131] and that the Phe4–Glu11 sequence, together with the Leu17–Lys28 hydrophobic region, of Aβ40/42 are responsible for the non-covalent interaction that occurs in the 17–21 region of the peptide (Leu-Val-Phe-Phe-Ala), which includes two Phe residues [197,198]. Interestingly, the Aβ sequence critical for amyloid fibrillization overlaps such OLE-binding regions. This finding supports the hypothesis that aromatic stacking or, more generally, hydrophobic interactions, would be the molecular mechanism underlying inhibition of amyloid aggregation by this polyphenol [197]. Hydrophobic interaction is probably also involved in the incorporation of olive polyphenols into LDL [199].

Fourth, in spite of the reduced bioavailability due to incomplete intestinal absorption, microbiota metabolism and biotransformation in tissue, plant, notably olive, polyphenols are indeed distributed throughout the organism and have been found in tissues, including the brain, further supporting their ability to positively interfere with pathological states and/or their prodromal conditions.

Further efforts are needed to mechanistically define the biochemical and biological activities of EVOO and olive polyphenols as well as the pharmacokinetics and pharmacodynamics underlying their effective doses in humans and the dose-dependence of their effects. The structure–activity relationship of olive polyphenols must still be deciphered as well; the latter could be the basis of engineering new drugs starting from the molecular scaffolds of these substances. Finally, more clinical trials are needed to overcome the limits of those currently reported and some of their conflicts. However, we do believe that the four points that we have extracted from the large body of scientific literature on OLE, HT and oleocanthal could be useful landmarks with which to orient future research and organize present and future data into a coherent frame.

## Figures and Tables

**Table 1:** EVOO (extra virgin olive oil) polyphenols, similarly to resveratrol, act as caloric restriction (CR) mimickers.

Name	Suggested Mechanism of Action	References
Resveratrol	Sirt activation	[25,27,30,36]
	↓	
	✓ Increased antioxidant defenses via Nrf-2 induction	[39]
	✓ Reduced inflammation via NFκB down regulation	[40]
	✓ Autophagy induction via AMPK activation	[45]
HT	Sirt activation	[26]
	↓	
	✓ Increased antioxidant defenses via Nrf-2 induction	[38]
OLE	Sirt activation	[26]
	↓	
	✓ Reduced adipogenesis via PPARγ inhibition	[41]
	✓ Autophagy induction via AMPK activation or direct modulation of insulin/IGF1/AKT and the mTOR pathways	[46,47,48]

**Table 2:** Effects of olive polyphenols suggesting their possible use in cancer prevention.

Name	Biological Activity	References
OLE	Reduced angiogenesis	[58]
	Apoptosis induction	[51,52,53,55,57,59,60]
	Cell cycle delay	[60]
	Metastasis prevention	[62]
	Recovered sensitivity to chemotherapeutics	[63,64]
	Reduced cell proliferation and viability	[55,56,65,66]
	Cytoskeleton disassembly	[67]
	Inhibition of epithelial-to-mesenchymal transition	[68]
	Activation of cellular stress-like genes	[69]
HT	Reduced angiogenesis	[58]
	Apoptosis induction	[59]
	Cell cycle arrest	[61]

**Table 3:** Effects of olive polyphenols suggesting their use in CVD (cardiovascular disease) prevention.

Name	Biological Activity	References
OLE	Reduction of lipid peroxidation	[77]
	Protection against ischemia/reperfusion-induced oxidative stress	[78]
	Total cholesterol and triglyceride reduction	[79]
	Inhibition of atherosclerosis	[83]
	Enhanced fat oxidation and optimized cardiac energy metabolism	[90]
	Inhibition of platelet aggregation	[92]
HT	Reduction of lipid peroxidation	[77]
	Protection against oxidative stress induced by ischemia/reperfusion	[78]
Tyrosol	Reduction of lipid peroxidation	[77]
	Protection against oxidative stress induced by ischemia/reperfusion	[78]
	Inhibition of atherosclerosis	[84]
EVOO high in polyphenols	Increase of HDL cholesterol and prevention of HDL oxidation	[81,82]
	Inhibition of atherosclerosis	[85]
	Reduction of post-prandial inflammation	[87]
Olive leaf extract	Reduction of post-prandial inflammation	[86]

**Table 4:** Effects of olive polyphenols suggesting their use in treating metabolic disorders.

Name	Biological Activity	References
OLE	Inhibition of intracellular triglyceride accumulation and adipocyte differentiation	[94,95]
	Inhibition of amylin aggregation into amyloid	[99]
	Reduction of glycaemia and increase of antioxidant defenses in animal models of diabetes	[100,101,102,112]
	Prevention of oxidative damage to pancreatic β-cells	[108]
	Liver protection against steatosis and oxidative damage	[109,113,114]
	Attenuation of visceral adiposity	[110]
	Inhibition of lipogenesis	[115,116]
	Prevention of NAFLD	[117,119]
HT	Inhibition of adipocyte differentiation	[95]
	Increased mitochondrial biogenesis and function	[96]
	Reduction of glycaemia and cholesterolemia and increase of antioxidant defenses	[100]
	Inhibition of lipogenesis	[115,116]
EVOO	Modulation of insulin sensitivity-related genes	[105]
	Increase of antioxidant defenses	[107]
Olive leaf extract	Inhibition of adipocyte differentiation, increased mitochondrial biogenesis and thermogenesis	[97]
	Reduction of glycaemia	[103]
	Reduced insulin resistance and improved pancreatic β-cell secretion	[104]
	Prevention of oxidative damage to pancreatic β-cells	[106,108]
	Attenuation of heart and liver modifications associated to metabolic syndrome	[111]
	Prevention of NAFLD	[117,118,120]

**Table 5:** Olive polyphenols and prevention of amyloid diseases.

Name	Biological Activity	References
OLE and OLE aglycone	Inhibition of Aβ toxic aggregation	[128]
	Inhibition of Tau toxic aggregation	[129]
	Reduced plaque load and health improvement in murine models of Aβ deposition	[47,48,134,137]
	Increase in the non-amyloidogenic Aβ cleavage by α-secretase	[136]
Oleocanthal	Inhibition of Tau toxic aggregation	[130]
	Reduction of Aβ oligomer toxicity	[131]
	Enhanced β-amyloid clearance	[135]

**Table 6:** Olive polyphenols in clinical trials and epidemiological studies.

Name	Biological Activity	References
EVOO	Protection against cognitive decline	[21,154,156]
	Protection against stroke	[155]
	Protection against CVD	[157,158,159,160]
	Protection against breast and colon cancers	[153,162]
	Reduced risk of T2DM	[163,164,165]
	Reduced post-prandial serum levels of glucose and LDL	[165]
	General reduction of inflammation markers	[169]
EVOO polyphenols	Reduction of triglycerides and glucose plasma levels and increase of nitric oxide, with lowered blood pressure	[158,159]
	Reduced oxidation of LDL	[160]
	General reduction of CVD risk factors	[161]
	Decrease of total cholesterol/HDL-cholesterol ratio and oxidative stress markers and protection against atherosclerosis	[81,82,165,167]
	Reduction of post-prandial inflammation and oxidative stress	[168,169,170,171]
